# HER2-positive gastric cancer: from targeted therapy to CAR-T cell therapy

**DOI:** 10.3389/fimmu.2025.1560280

**Published:** 2025-03-13

**Authors:** Qiangzu Shao, Junge Deng, Haoran Wu, Zeping Huang

**Affiliations:** ^1^ The Second Hospital & Clinical Medical School, Lanzhou University, Lanzhou, China; ^2^ Key Laboratory of the Environmental Oncology of Gansu Province, The Second Hospital & Clinical Medical school, Lanzhou, China

**Keywords:** gastric cancer, HER2, targeted therapy, cell therapy, drug resistance

## Abstract

Gastric cancer (GC) ranks as the fifth most prevalent cancer on a global scale, with HER2-positive GC representing a distinct subtype that exhibits more intricate biological characteristics. Conventional chemotherapy typically exhibits restricted efficacy in the management of HER2-positive GC. In light of the incessant advancement in molecular targeted therapies, targeting HER2 has emerged as a promising therapeutic approach for this subtype. The advent of antibody-drug conjugates (ADCs) and chimeric antigen receptor T-cell therapy (CAR-T) has furnished novel treatment alternatives for HER2-positive GC. Nevertheless, owing to the pronounced heterogeneity of GC and the complex tumor microenvironment, drug resistance frequently emerges, thereby substantially influencing the effectiveness of HER2-targeted therapy. This article comprehensively summarizes and deliberates upon the strategies of HER2-targeted therapy as well as the underlying resistance mechanisms.

## 1 Introduction

**Figure 1:**
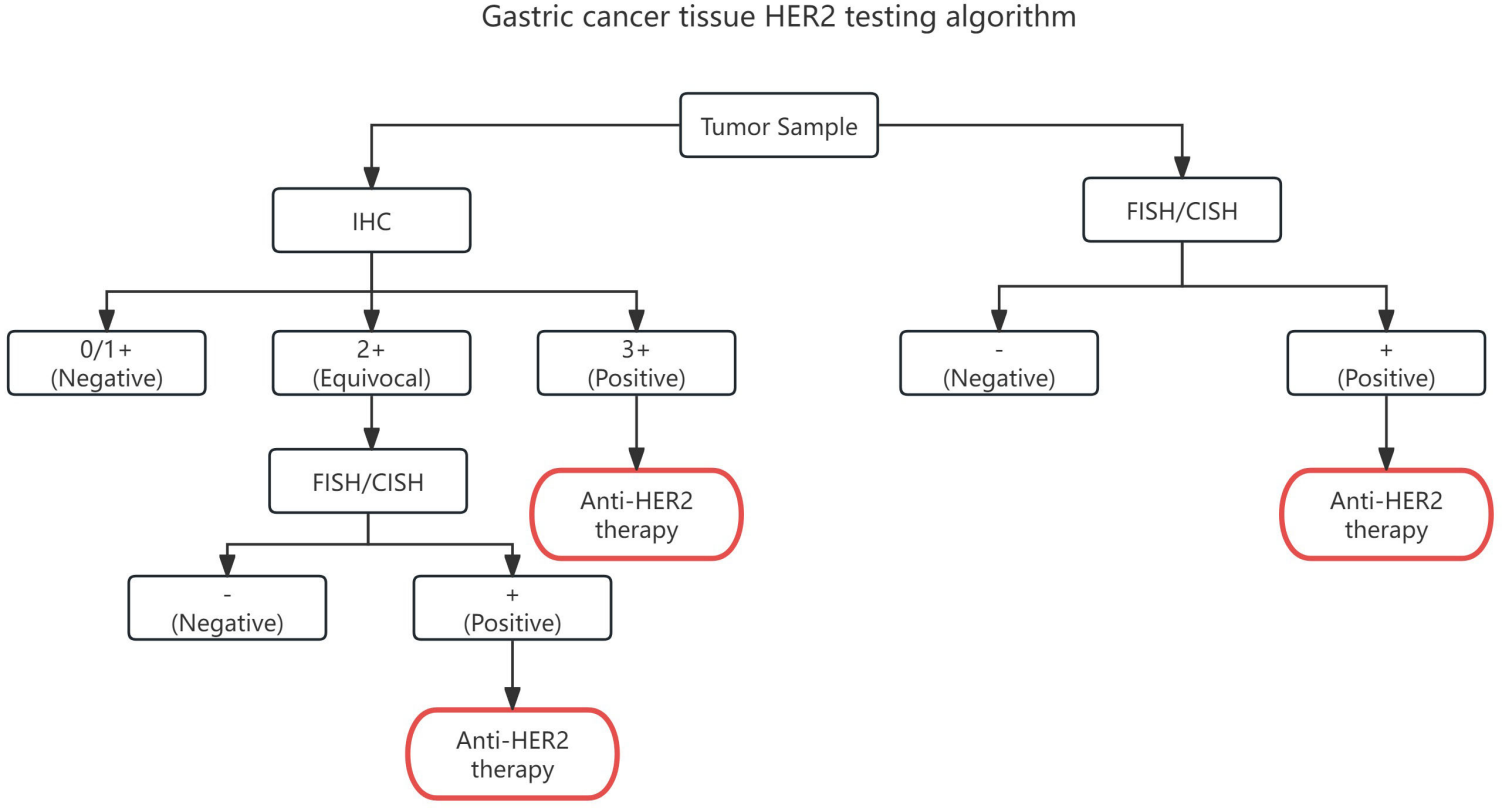
Gastric cancer tissue HER2 testing algorithm.

In accordance with the global cancer statistics published by GLOBOCAN in 2022 (1), GC ranks as the fifth leading cause of cancer-related mortality globally and the fifth most prevalent malignant neoplasm. Significant geographical disparities in GC incidence are evident, with the highest rates observed in East Asian countries (1). Despite the incidence of GC in China is declining, it remains among the top three cancers in terms of incidence and mortality nationwide (2).In the pathogenesis and advancement of GC, chronic Helicobacter pylori infection, smoking, alcohol consumption, a high-salt diet, elevated body mass index, and Epstein-Barr virus infection assume significant roles (3, 4). The overexpression of human epidermal growth factor receptor 2 (HER2) in GC is intimately correlated with tumor progression and poor prognosis (5). Approximately 10-20% of GC patients display HER-2 overexpression, rendering it a valuable prognostic and predictive biomarker (6). HER2 expression is divided into different states. Immunohistochemistry (IHC) is used to detect HER2 protein, fluorescent *in situ* hybridization (FISH) or chromogenic *in situ* hybridization (CISH) is used to detect gene amplification. HER2 overexpression can be classified as IHC0 (Negative), IHC1+ (negative), IHC2+ (suspicious), or IHC3+ (positive), and samples with IHC2+ values should undergo another FISH or CISH test(**Figure 1**) (7, 8).

## 2 HER2-related signaling pathways

**Figure 2:**
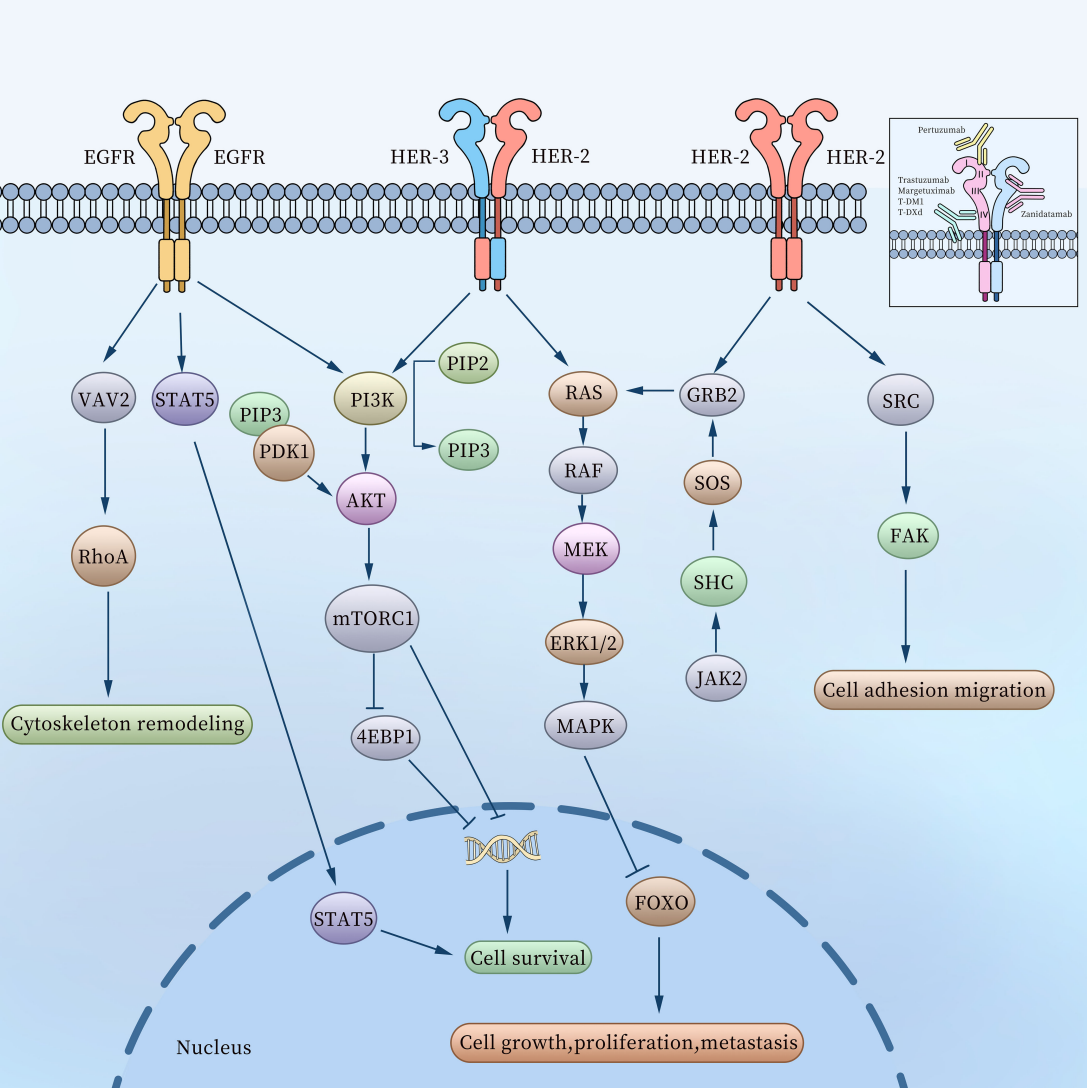
The related molecular mechanisms of the EGFR family in gastric cancer and binding sites of HER2-targeting antibodies.

HER2, encoded by ErbB2 gene located on human chromosome17 (9), is a transmembrane receptor with intrinsic tyrosine kinase activity. As a member of the epidermal growth factor receptor (EGFR) family, HER2 belongs to a group that encompasses HER1 or EGFR, HER2, HER3, and HER4. Each receptor in this family comprises three key components: an extracellular ligand-binding domain, a transmembrane domain, and an intracellular activation domain (10, 11). The binding of various ligands to the extracellular domain triggers a series of signal transduction pathways (**Figure 2**), which play pivotal roles in regulating tumor cell growth, apoptosis, adhesion, migration, and differentiation (12).

The HER2 monomer lacks ligand-binding activity and requires dimerization to become functionally active (13), thus forming HER2-HER2 homodimers, HER2-HER3 and EGFR-HER2 heterodimers. Among these, the HER2-HER3 heterodimer is the most potent HER signaling receptor and plays a vital role in the initiation and progression of cancer (14). The HER2-HER2 homodimer promotes cell adhesion, migration, growth, proliferation, and metastasis by activating signaling pathways such as SRC-FAK (15), GRB2-SOS-JAK2 (16), and RAS-MEK-MAPK (17) within GC cells. Meanwhile, the HER2-HER3 heterodimer transmits signals through the RAS-MEK-MAPK and PI3K-AKT pathways, further driving tumor progression (18). After EGFR binds to HER2 to form EGFR-HER2 heterodimers, it triggers receptor conformational changes, activates intracellular tyrosine kinase domains, significantly enhances downstream signals (such as MAPK, PI3K-AKT pathways), and promotes tumor cell proliferation, survival and metastasis (19). In addition, it has been found that elevated EGFR expression levels can promote the formation of EGFR-HER2 heterodimers and inhibit the internalization and efficacy of antibody-drug conjugates (ADCs), and the internalization and efficacy of ADCs can be restored after knockout or drug-targeted EGFR (20).

## 3 Targeted HER2 therapy in GC drug research

**Figure 3:**
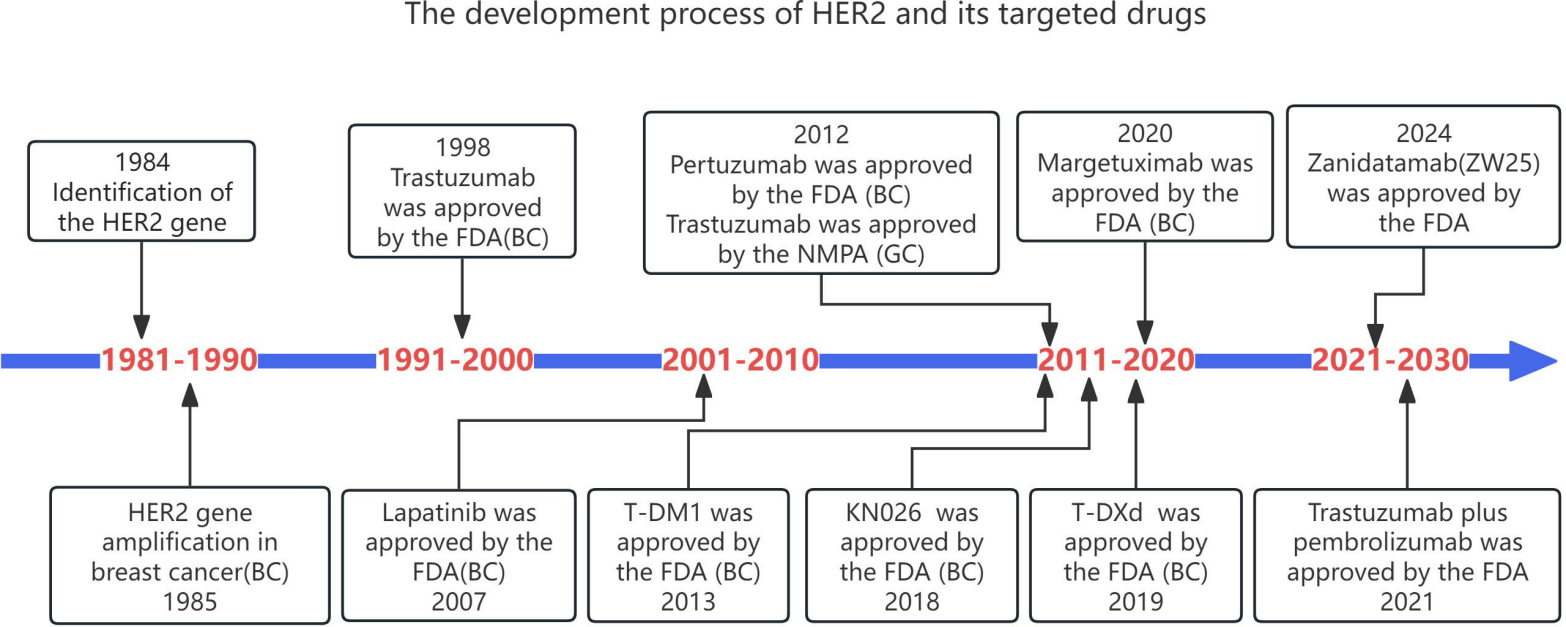
The development process of HER2 and its targeted drugs.

Over the past three decades, within the realm of solid tumors exemplified by gastric cancer and breast cancer, HER2 has emerged as a key target for novel drugs, including tyrosine-kinase inhibitors (TKIs), antibody-drug conjugates (ADCs), and dual-antibody agents. HER2 also occupies key target positions in gastric cancer(**Figure 3**). However, looking at the development history of HER2 and its targeted drugs, there are only a few successful drugs. Trastuzumab is the first proven first-line targeted therapeutic drug for gastric cancer, with good results in the treatment of advanced GC. Clinical efficacy is the first-line standard therapy for advanced HER-2-positive GC (21). Numerous novel HER2-targeted drugs are currently under development, including Pertuzumab, Margetuximab, Zanidatamab (ZW25), KN026 (a HER2 bispecific antibody),Tyrosine kinase inhibitors (TKIs),as well as Antibody Drug Conjugates like Trastuzumab Emtansine (T-DM1), Trastuzumab Deruxtecan (T-DXd), and Disitamab Vedotin (RC48).These drugs all exhibit certain therapeutic efficacies in the treatment of gastric cancer (**Table 1**).

**Table 1:** HER2-targeted drugs involved in clinical trials for the treatment of gastric cancer.

Category	Agents	Clinical trial/NCT number	Phase	Study arms	Enrolled population	Status	Key results
HER-2 monoclonal antibody	Trastuzumab	TOGA	III	Cisplatin/fluoropyrimidine + trastuzumab vs. cisplatin/fluoropyrimidine	HER2-positive advanced GC/GEJ	Completed	mOS: 13.8 vs. 11.1months mPFS: 6.7 vs. 5.5months
		HERXO	II	Capecitabine+oxaliplatin (XELOX)+trastuzumab	HER2-positive advanced GC/GEJ	Completed	mOS: 13.8 months mPFS: 7.1 months
		PANTHERA	Ib/II	Pembrolizumab+trastuzumab+capecitabine+cisplatin	HER2-positive advanced GC/GEJ	Completed	ORR: 76.7% mOS: 19.3 months mPFS: 8.6 months
		PETRARCA	II	Trastuzumab+pertuzumab+5-FU,leucovorin,docetaxel,oxaliplatin(FLOT) vs. FLOT regimen	HER-2-positive,locally advanced,resectable GC/GEJ adenocarcinoma	Completed	PCR:35%vs.12% The 24-months survival rate: 84%vs.77%
		JACOB	III	Trastuzumab+pertuzumab+chemotherapy vs.chemotherapy	HER2-positive advanced GC/GEJ	Completed	mOS: 17.5 vs. 14.2months mPFS: 8.5 vs. 7.2months
	Pertuzumab	JOSHUA	II	Pertuzumab (840mg for cycle 1 and 420mg q3w for cycles 2–6 vs.pertuzumab 840mg q3w for six cycles)+trastuzumab+capecitabine+cisplatin	HER2-positive advanced GC	Completed	PR: 88% vs. 55% SD: 14% vs. 27%
		JACOB	III	Pertuzumab+chemotherapy+trastuzumab vs.chemotherapy+trastuzumab	HER2-positive metastatic GEJ/GC	Completed	mOS:18.1 vs. 14.2 months mPFS:8.5 vs. 7.2 months
	Margetuximab	NCT01148849	I	Margetuximab(Slingle-agent)	HER2-positive BC/GC	Completed	PR:12% mPFS: 15.1 vs. 21.1 weeks
		NCT01195935	III	Margetuximab+chemotherapy vs.chemotherapy+trastuzumab	HER2-positive BC	Completed	mPFS: 5.8 vs. 4.9 months ORR: 22% vs.16%
		MAHOGANY (NCT04082364)	II/III	Margetuximab+retifanlimab vs. historical data of chemotherapy+trastuzumab	HER2-positive/PD-L1-positive and non-microsatellite instability-high GEJ/GC	Completed	ORR:53% MDOR: 10.3 months DCR: 73%
Small molecule tyrosine kinase inhibitor	Lapatinib	NCT00103324	II	Lapatinib	HER2-positive metastatic GEJ/GC	Completed	mOS: 4.8 months
		TyTAN (NCT00486954)	II	Lapatinib plus paclitaxel vs. paclitaxel	HER2-positive advanced GC	Completed	mOS: 11.0 vs. 8.9 months mPFS: 5.4 vs.4.4months
		LOGiC (‘NCT00680901)	III	Lapatinib plus capecitabine and oxaliplatin vs. placebo plus capecitabine and oxaliplatin	HER2-positive advanced GC	Completed	mOS: 12.2 vs. 10.5 months mPFS:6.0 vs.5.4months
	Afatinib	NCT01522768	II	Afatinib monotherapy	HER2-positive GEJ/GC,following failure of at least one trastuzumab/chemotherapy regimen	Completed	ORR:10% mOS:7.0 months mPFS: 2.0 months
	Neratinib	NCTO1953926	II	Neratinib monotherapy	Solid tumors with somatic activating HER-2 mutations	Recruiting	NA
Antibody-drug conjugate	Trastuzumab emtansine (T-DM1)	GATSBY	II/III	Taxane vs. T-DM1	HER2-positive, unresectable advanced GC who progressed on 1st-line fluoropyrimidine+platinum ± HER2-targeted therapy	Completed	mOS: 7.9 vs. 8.6 months mPFS: 2.7 vs. 2.9 months
	Trastuzumab deruxtecan (T-DXd)	DESTINY-Gastric01 (NCT03329690)	II	T-DXd vs. physician’s choice of chemotherapy	HER2-positive GC/GEJ	Completed	mOS: 12.5 vs. 8.4 months mPFS: 5.6 vs. 3.5 months
	RC48-ADC	RC48–008 (NCT04714190)	III	RC48-ADC monotherapy	HER2-overexpressing locally advanced or metastatic GC	Completed	ORR: 18.1% mOS: 7.6 months mPFS: 3.8 months
		NCT04280341	I	RC48 plus toripalimab	HER2-positive advanced GC/GEJ	Completed	ORR:43% mPFS:6.2 months mOS: 16.8 months

These HER2-targeting antibodies exhibit unique binding specificities. For instance, Trastuzumab, Margetuximab, T-DM1, and T-DXd bind to extracellular domain IV of HER2, while Pertuzumab binds to extracellular domain II. Zanidatamab binds to both extracellular domains II and IV of HER2. The molecular activation pathways and binding sites of HER2-targeting antibodies are illustrated in **Figure 2**.

### 3.1 Monoclonal antibodies targeting HER2

Monoclonal antibodies targeting HER2 recognize HER2 antigens via their Fab segment and attach to immune cells through their Fc segment, thereby performing anti-tumor functions by means of antibody-dependent cell cytotoxicity (ADCC), antibody-dependent cellular phagocytosis (ADCP), and complement-dependent cytotoxicity (CDC) (22, 23). In comparison with other targeted drugs, monoclonal antibodies targeting HER2 possess greater specificity in anti-tumor activities. Nevertheless, due to its relatively large molecular weight, the antibody is incapable of traversing the blood-brain barrier, thus exhibiting limitations in the treatment of patients with brain metastases.

#### 3.1.1 Trastuzumab

Trastuzumab is a recombinant humanized monoclonal IgG antibody that specifically targets the HER2 receptor, binds to the HER2 extracellular domain IV, impedes cell proliferation signal transduction, and exhibits an ADCC effect (23). The TOGA trial demonstrated that in comparison to chemotherapy alone, the overall survival (OS) of patients receiving chemotherapy in combination with trastuzumab was remarkably enhanced (11.1 months vs 13.8 months) (24). This finding was further supported by a Japanese clinical trial (25). In addition to effectively augmenting OS, the combination of chemotherapy and trastuzumab treatment can also ameliorate the quality of life of patients, and the recovery and adjustment time of toxic side effects is also shorter than that of chemotherapy alone (26). In a clinical study evaluating trastuzumab combined with capecitabine and oxaliplatin for advanced gastric cancer (GC), the combination treatment group showed notable efficacy, with an OS of 13.8 months compared to 7.1 months for chemotherapy alone (27, 28). Several other studies have also substantiated the efficacy and safety of trastuzumab in the treatment of HER-2 positive GC (29, 30).

With the advancement of tumor immunotherapy, the combination of trastuzumab and immune checkpoint inhibitors has emerged as a highly promising treatment modality. A phase II trial indicated that the combination of trastuzumab, nivolumab (a PD-1 inhibitor), and FOLFOX chemotherapy significantly improved overall survival (OS) in HER2-positive gastric cancer (GC) patients (21.8 months vs. 16.4 months) (31). Simultaneously, pembrolizumab (PD-1 inhibitor) can also be safely utilized in combination with trastuzumab and chemotherapy (32). In a phase III trial of trastuzumab and chemotherapy, the objective response rate (ORR) of patients was increased by 22.7% (77.4% vs 51.9%) and the complete response rate (CRR) was increased by 8.2% (11.3% vs 3.1%) after pembrolizumab was combined with trastuzumab and chemotherapy. These interim results uniformly suggest that the efficacy of this combination regimen, with further results on overall survival (OS) and progression-free survival (PFS) eagerly awaited (33).

#### 3.1.2 Pertuzumab

Pertuzumab is a second-generation anti-HER2 drug with a distinct mechanism of action compared to trastuzumab. Pertuzumab targets the extracellular domain II of HER2, effectively inhibiting the formation of HER2 dimers, particularly HER2-HER3 heterodimers, and blocking downstream signal transduction pathways (34).Studies have revealed that, in comparison with the combination of trastuzumab and chemotherapy, the addition of pertuzumab can significantly enhance the prognosis of patients with advanced HER2-positive breast cancer (BC) (35). Another study demonstrated that pertuzumab significantly prolonged the PFS of patients with HER2-positive BC (36). However, in the treatment of HER2-positive GC, the combination of pertuzumab with trastuzumab and chemotherapy did not result in a significant improvement in patient survival (37). This might be associated with the tumor heterogeneity between BC and GC. Consequently, additional studies are requisite to further ascertain the efficacy of pertuzumab in HER2-positive GC.

#### 3.1.3 Margetuximab

Margetuximab represents a novel Fc segment-optimized anti-HER2 monoclonal antibody (38). Unlike trastuzumab, margetuximab has modified five amino acid residues (L235V/F243L/R292P/Y300L/P396L) within the Fc region. This modification not only augments the binding with the activating Fc receptor FcγRIIIA-CD16A but also diminishes the binding with the inhibitory Fc receptor FcγRIIB-CD32B, thereby significantly enhancing the response rate of margetuximab (39). Additionally, CD16A is a crucial receptor that mediates ADCC. Consequently, margetuximab is more efficacious than trastuzumab with a wild-type Fc domain and exhibits a more potent ADCC effect, thus manifesting a stronger cytotoxic effect on tumor cells (40, 41). In a phase Ib/II clinical trial, the combination of margetuximab and pembrolizumab demonstrated synergistic anti-tumor activity, especially in tumors with low HER2 expression or in patients with low CD16A binding alleles (42). Another phase II trial of margetuximab and pembrolizumab as second-line treatment for HER2-positive gastric cancer (GC) demonstrated notable efficacy, with an objective response rate (ORR) of 28.2%, a disease control rate (DCR) of 63.4%, a median progression-free survival (mPFS) of 4.3 months, and a median overall survival (mOS) of 13.9 months, accompanied by good safety and tolerability (43). The aforementioned studies have indicated that the combination of margetuximab and pembrolizumab can cooperatively enhance the activity of anti-tumor drugs. The CMAHOGANY trial combined retifanlimab (PD-1 inhibitor) with margetuximab for the first-line treatment of HER2-positive GC, and the results exhibited an ORR of 53% and a DCR of 73% (44). Currently, a phase II/III randomized trial is underway to evaluate the efficacy of margetuximab combined with retifanlimab (PD-1 antibody) (with or without chemotherapy) and margetuximab combined with tebotelimab (PD-1 antibody) in patients with HER2-positive GC or gastroesophageal junction cancer (45).

#### 3.1.4 ZW25

ZW25 is a bispecific antibody capable of simultaneously binding to the extracellular domains II and IV of HER2. In comparison to trastuzumab or pertuzumab, ZW25 exhibits stronger anti-tumor activity, effectively suppresses HER2 signaling, and enhances immune system activation and antibody-dependent cellular cytotoxicity (ADCC) effects (46, 47). In a clinical study of HER2-positive GC, the combination of ZW25 and chemotherapy yielded favorable outcomes (ORR: 54%, DCR: 79%) (48). Another clinical trial determined that ZW25 has a significant anti-tumor effect in the treatment of HER2-expressing or amplified cancers (49). A phase II study evaluating ZW25 in combination with chemotherapy demonstrated notable improvements in patient survival, achieving an ORR of 79%, a duration of response (DOR) of 20.4 months, and progression-free survival (PFS) and overall survival (OS) of 12.5 months and not yet reached, respectively (50). The most common treatment-related adverse event associated with ZW25 is diarrhea, necessitating prophylactic use of antidiarrheal medications during clinical trials (51). A phase III trial comparing ZW25 in combination with chemotherapy, ZW25 in combination with tislelizumab and chemotherapy, and trastuzumab in combination with chemotherapy is currently in progress, and promising research results are anticipated.

#### 3.1.5 KN026

KN026 is a novel bispecific antibody targeting HER2. It binds to both extracellular domains II and IV of HER2. KN026 has demonstrated high affinity and potent tumor-suppressing capabilities in HER2-positive tumor cell lines. Additionally, it exerts inhibitory effects on tumors with low HER2 expression and trastuzumab-resistant cell lines (52).In a study involving patients with previously treated advanced HER2-positive gastric cancer, the ORR in the HER2 high-expression cohort was 56%, with a remission duration of 9.7 months. In the HER2 low-expression cohort, the ORR was 14%. The most prevalent adverse events were related to gastrointestinal disorders, and no drug-related fatalities occurred. This indicates that KN026 has a favorable safety profile along with potent anti-tumor activity (53).Clinical investigations have shown that the anti-tumor activity of KN026 surpasses that of trastuzumab or pertuzumab used alone. Moreover, its anti-tumor activity is comparable to or better than the combination of trastuzumab and pertuzumab (54).The results of a phase II study on the combination of KN026 and docetaxel for the treatment of HER2-positive breast cancer demonstrated good efficacy and a manageable safety profile in the first-line treatment of HER2-positive recurrent/metastatic breast cancer (55).

#### 3.1.6 TKIs

TKIs are a class of medications that impede cell signaling by suppressing the activity of tyrosine kinases. They are predominantly utilized for treating a diverse range of cancers. TKIs inhibit tyrosine kinase activity via multiple mechanisms, such as obstructing ATP-binding sites, inhibiting phosphorylation reactions, and blocking signaling pathways (56). These inhibitors can be categorized into various types, including those targeting EGFR, VEGFR,HER2, ALK,RET, MET, MEK,FGFR (57). Lapatinib was initially approved in 2007 as a small-molecule tyrosine-kinase inhibitor targeting both EGFR and HER2 for the treatment of HER2-positive breast cancer (58). In the treatment of HER2-positive gastric cancer, a comparison of the combination of lapatinib with oxaliplatin and capecitabine against capecitabine and oxaliplatin alone revealed OS values of 10.5 months and 12.2 months, and PFS values of 6.0 months and 5.4 months in the respective groups (59). In another study, when comparing lapatinib plus paclitaxel to paclitaxel alone, the OS was 11.0 months and 8.9 months, and the PFS was 5.4 months and 4.4 months, respectively (60). These two studies indicated that the combination of lapatinib with chemotherapy drugs did not significantly enhance the OS of HER2-positive gastric cancer patients. This could potentially be ascribed to lapatinib-related toxicity and patient-specific demographic factors, including age and geographical region. Although afatinib and tucatinib have demonstrated some antitumor activity in HER2-positive gastric cancer patients (61, 62), additional research is necessary to precisely determine their efficacy.

### 3.2 ADCs

**Figure 4:**
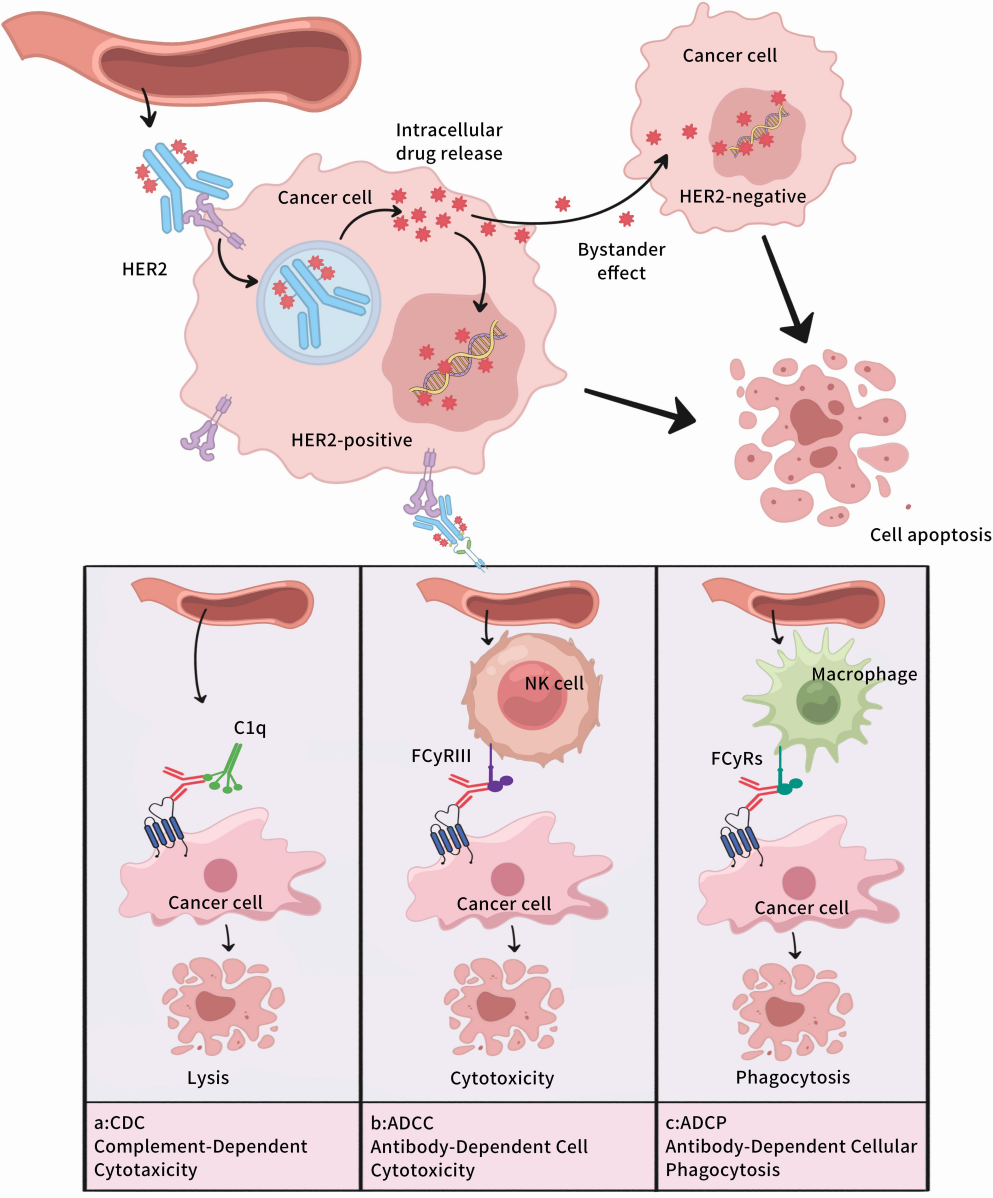
Mechanism of Action of Antibody-Drug Conjugates. **(A)**Complement Dependent Cytotoxicity,CDC;**(B)** Antibody dependent cell mediated cytotoxicity,ADCC;**(C)** Antibody-Dependent Cellular Phagocytosis,ADCP.

Antibody-drug conjugates (ADCs) represent a class of targeted therapies composed of monoclonal antibodies directed against specific antigens, small molecule cytotoxic drugs, and chemical linkers(**Table 2**) (63). By leveraging blood circulation, ADCs precisely deliver their therapeutic payload to the target site, where they exert dual functions: the potent cytotoxic effects of small-molecule chemotherapy drugs and the tumor-targeting specificity of antibody-based therapies (64), as depicted in **Figure 4**.

**Table 2:** Antibody-drug conjugates (ADCs) drugs for HER2-targeted therapy.

ADC	Target	Antibody	Payload	Linker	DAR	Year
Trastuzumab Emtansine(T-DM1)	HER-2	Trastuzumab	DM1	Non-cleavable SMCC linker	3.5	2013
Trastuzumab Deruxtecan (T-DXd)	HER-2	Trastuzumab	DXd	Cleavable GGFG linker	7-8	2021
Disitamab Vedotin(RC48)	HER-2	Hertuzumab	MMAE	Cleavable vc-PABC linker	4	2021
Trastuzumab Duocarmazine(SYD985)	HER-2	Trastuzumab	Seco-DUBA	Cleavable vc linker	2.7	2017
ARX-788	HER-2	Anti-HER2 mAb(ARX269)	MMAF	Non-cleavable linker conjugated to pAcF	1.9	2021
A166	HER-2	Anti-HER2 mAb	Duostatin-5	Cleavable vc linker	N/A	2021
MRG002	HER-2	Anti-HER2 mAb	MMAE	Cleavable vc linker	3.8	2020
ALT-P7	HER-2	Trastuzumab biobetter(HM2)	MMAE	Cleavable cysteine-containing peptide linker	2	2020
GQ1001	HER-2	Trastuzumab	DM1	N/A	N/A	2022
SBT6050	HER-2	Anti-HER2 mAb	Toll-like receptor 8 agonist	N/A	N/A	2020
PF-06804103	HER-2	Trastuzumab-derived Ab	Aur-0101	Valine-citrulline linker	4	2020

#### 3.2.1 T-DM1

T-DM1 is an antibody-drug conjugate (ADC) comprising trastuzumab, a maytansine derivative (DM1, a tubulin inhibitor), and a thioether linker, with a drug-antibody ratio (DAR) of 3.5 (65). Serving as a targeted delivery system, T-DM1 transports DM1 to tumor cells with high HER2 expression through cell membrane receptor-mediated endocytosis. It then binds to tubulin, prevents microtubule polymerization and formation, and halts the cell cycle at the metaphase of mitosis. Meanwhile, trastuzumab binds to the HER2 extracellular domain IV, blocks the PI3K/AKT pathway, inhibits tumor cell proliferation, and the Fc segment of trastuzumab can also activate ADCC and induce apoptosis of tumor cells (66).

T-DM1 is the first ADC for solid tumors and was approved by the US Food and Drug Administration (FDA) in 2013 for the treatment of HER2-positive advanced breast cancer (67). In the KATHERINE-PHASE III STUDY, T-DM1 reduced the risk of disease recurrence or death by 50% in patients with residual HER-2-positive breast cancer after neoadjuvant therapy (68). In patients with HER2-positive advanced breast cancer who had previously been treated with paclitaxel and trastuzumab, T-DM1 demonstrated PFS of 9.6 months and OS of 29.9 months (69). Given its favorable therapeutic effect on breast cancer, its efficacy in GC has also attracted significant attention. Although T-DM1 demonstrated potent suppression of GC cell proliferation in preclinical trials, it did not yield a significant survival benefit in subsequent clinical trials (70). The GATSBY trial, which assessed the efficacy of T-DM1 in patients with HER2-positive GC, reported a median overall survival of 11.8 months in the T-DM1 group compared to 10.0 months in the paclitaxel chemotherapy group. The difference in survival between the two groups was not statistically significant, indicating that T-DM1 did not offer a clear survival advantage (71). Consequently, large-scale clinical trials are required to comprehensively explore the potential of T-DM1 as a viable treatment option for advanced GC.

#### 3.2.2 T-DXd

T-DXd is an antibody-drug conjugate (ADC) composed of trastuzumab, topoisomerase I inhibitor, and cleavable peptide linker (72).This design enables the precise delivery of its cytotoxic payload into HER2-positive tumor cells, minimizing damage to normal cells (73). In contrast to T-DM1, T-DXd has DAR of 8, exhibits a robust bystander effect, and also demonstrates favorable antitumor activity in HER2-low and negative GC patients (74).

T-DXd significantly enhanced the RR (51% vs 14%) and prolonged OS(12.5 months vs 8.4 months) in patients with HER2-positive GC when compared with conventional chemotherapy (75). In a phase II trial, the outcomes of T-DXd were as follows: ORR:25%, DCR:42%, and mOS:7.6 months in HER2-positive patients who had received two or more systemic chemotherapy treatments (76). The DESTINY-Gastric02 results indicated that T-DXd alone was effective as a second-line treatment for patients with HER2-positive advanced GC (ORR: 42.0% and CRR: 5%) (77). A study demonstrated that T-DXd has significant efficacy and safety in the treatment of HER2-positive GC (78). Another Phase II clinical study evaluated T-DXd in HER2-positive solid tumors(mDOR: 11.3 months, mPFS: 6.9 months, mOS: 13.4 months) (79). Although T-DXd has exhibited good therapeutic efficacy, a substantial number of studies are still necessary to confirm the safety and efficacy of T-DXd monotherapy or its combination with chemotherapy and immunotherapy.

#### 3.2.3 RC48

RC48 is a first-in-class ADC drug in China. It is composed of Hertuzumab coupled to monomethyl auristatin E (MMAE) through a cleavable linker and exerts anti-tumor effects by modulating the proliferation, differentiation, apoptosis, and migration of tumor cells (80). Compared with Trastuzumab, RC48 has a higher affinity for HER2-positive cells and a stronger ADCC effect (81). RC48 has demonstrated a favorable safety profile and notable antitumor activity in studies involving HER2-positive gastric cancer (GC) (82). Additionally, when co-cultured with HER2-positive and HER2-negative GC cells, RC48 exhibited cytotoxic effects on HER2-negative cells as well, highlighting its strong bystander effect (83).

RC48 was capable of significantly inhibiting the proliferation of HER2-positive GC cells in a dose-dependent manner and exhibited potent antitumor activity in a trastuzumab-resistant xenograft tumor model. This might be related to the RC48 bystander effect (84). Wang applied RC48 in combination with toripalimab (PD-1 inhibitor) for the treatment of GC patients (85). The results showed that the ORR was 43%, the mPFS was 6.2 months, and the mOS was 16.8 months. Clinical benefits were observed in both HER2-positive and HER2-negative populations. The combination of RC48 and toripalimab had a manageable safety profile and good clinical efficacy. A phase II study in advanced GC yielded similar results (86), with an ORR of 24.8%, mPFS of 4.1 months, and mOS of 7.9 months. The study found that RC48 had significant anti-tumor activity and a good safety profile in HER2-positive GC patients, and its effect was not entirely dependent on HER2 expression (82). Meanwhile, the combination of RC48 with other targeted drugs or chemotherapeutic agents might be a promising therapeutic strategy for the treatment of advanced HER2-positive GC and is expected to achieve more favorable results in the future.

#### 3.2.4 Combination treatment of ADC

In recent years, gastric cancer treatment has entered the era of precision medicine, and the advent of ADCs has revolutionized the treatment paradigm of solid tumors. Nevertheless, the majority of patients fail to achieve sustained disease control following ADC administration and develop resistance to ADCs. Consequently, combination therapy has emerged as the primary approach to optimizing the therapeutic efficacy of ADCs. Such combination therapies encompass combinations with chemotherapy, targeted therapy, immunotherapy, among others.

The combination of ADCs and chemotherapy has been demonstrated to be a widely-accepted strategy for overcoming drug resistance and enhancing therapeutic efficacy (87). The selection of chemotherapeutic agents can influence the levels of surface antigens targeted by ADCs. For instance, gemcitabine can upregulate the expression of HER2 on pancreatic cancer cells by 14.81-fold. This implies that the combination of T-DM1 and gemcitabine may enhance the effective binding of T-DM1 to HER2, thereby augmenting the combined treatment effect (88). However, the combination of ADCs with chemotherapy may exacerbate adverse reactions. Research has indicated that when T-DM1 is combined with docetaxel in HER2-positive breast cancer patients, dose-related toxicity and adverse events occur in 80% of patients, including leukopenia, epistaxis, nausea, and diarrhea (89). Additionally, the combination of T-DM and capecitabine has led to an increased drug discontinuation rate without a significant improvement in clinical efficacy (90). In metastatic HER2-positive gastric cancer patients, the combination of T-DXd with 5-fluorouracil or capecitabine is associated with dose-related oral mucositis and a higher incidence of adverse events (91). Overall, these study results suggest that the toxicity significantly increases when ADCs are combined with conventional chemotherapy. This may be attributed to the elevated toxicity resulting from the off-target effects and extratumoral actions of the ADC payload.

Targeted therapies have demonstrated clinical safety and efficacy. However, the efficacy of their combination with ADCs remains relatively poorly understood. The combined use of T-DM1 and pertuzumab has shown synergistic activity in cell culture models and was well-tolerated in phase Ib and phase II studies (92, 93). Bordeau et al. discovered that in a mouse model of NCI-N87 subcutaneous xenograft tumors, the co-administration of an anti-trastuzumab single-domain antibody (1HE) and T-DM1 significantly enhanced the efficacy of T-DM1 and prolonged the median survival period (94). The results of several phase III clinical trials indicate that the T-DM1 plus pertuzumab regimen reduces the incidence of adverse events. Compared with the chemotherapy plus trastuzumab and pertuzumab regimens, this regimen exhibits superior clinical efficacy (95–97). As a small-molecule tyrosine kinase inhibitor targeting HER2, tucatinib significantly enhances its efficacy when combined with trastuzumab or docetaxel, effectively improving the partial and complete tumor regression rates (98). Several other studies have also shown that the combination of T-DM1 and tucatinib can enhance the efficacy of T-DM1 (99, 100). The co-administration of osimertinib (an EGFR inhibitor) and T-DM1 facilitates the enhancement of antitumor effects, and T-DM1 can overcome osimertinib resistance in an EGFR-mutated non-small-cell lung cancer model (101).

The combination of ADCs with immunotherapy can synergistically enhance efficacy, and the selection of low-toxic and well-tolerated ADCs can lead to better therapeutic outcomes. There is a growing body of evidence suggesting that the combination of immunotherapy with ADCs has improved antitumor effects (102). Some researchers have utilized low-dose ADCs as immune stimulators in animal experiments and found that ADCs exhibit a more potent effect in animal models with normal immune function compared to immunodeficient models, indicating that ADCs possess significant immunomodulatory capabilities and can enhance the efficacy of immunotherapy (103, 104). A prospective clinical trial (KATE2) in advanced HER2-positive breast cancer evaluated the efficacy of the combined treatment of ADC and immune checkpoint inhibitors (105). The efficacy of T-DM1 combined with atezolizumab was compared to that of T-DM1 placebo. The study revealed that the mPFS of patients in the atezolizumab group was 8.2 months, while that in the placebo group was 6.8 months. In terms of safety, serious adverse events occurred in 43 out of 132 patients treated with atezolizumab and 13 out of 68 patients treated with placebo (19%). Another single-arm study employed T-DM1 in combination with pembrolizumab for the treatment of HER2-positive metastatic breast cancer. Twenty patients received the combination therapy, with an mPFS of 9.6 months and an ORR of 20%, without dose-related toxicity (106).

In summary, ADCs have demonstrated significant efficacy as monotherapy in many cancers. Their combination with chemotherapy or targeted therapy has shown certain advantages, yet issues such as adverse reactions and drug resistance still persist. Although the combination of ADCs with ICIs holds promising prospects, further research is required to elucidate the survival improvement and biological mechanisms in solid tumors.

The aforementioned HER2-targeted drugs have demonstrated favorable therapeutic effects in clinical applications. Compared to traditional chemotherapy, targeted drugs exhibit high specificity, fewer side effects, and better efficacy. Moreover, targeted drugs can be combined with chemotherapy or immunotherapy to further enhance their efficacy. However, targeted therapy drugs are restricted to HER2-positive gastric cancer patients and are ineffective in HER2-negative patients. Simultaneously, during the course of targeted drug therapy, the emergence of primary or secondary drug resistance is a key factor influencing patient efficacy. Addressing the issue of drug resistance and improving drug sensitivity are the focal points in the development of HER2-targeted therapy drugs.

## 4 CAR-T therapy

Chimeric antigen receptor T (CAR-T) represents a novel type of precision-targeted immunotherapy for tumor treatment. In this approach, CAR-T cells are genetically engineered either from patients or donors to acquire the capacity to recognize and efficiently eliminate tumor cells. Subsequently, these engineered cells are infused back into the patient’s body to initiate an effective anti-tumor immune response, thereby achieving the objective of treating malignant tumors (107). Unlike conventional T cells, CAR-T cells function independently of major histocompatibility complexes (MHCs), enabling them to recognize a wide range of T cell tumor-associated antigens (108). CAR-T cells are composed of four components: the antigen recognition region, the hinge chain, the transmembrane region, and the intracellular signaling region. The antigen recognition region is typically constituted by a signal peptide and a single-chain antibody (scFv) fragment that recognizes tumor-specific antigens. This region can identify and bind to antigens on the surface of tumor cells and connect the antigen recognition region to the transmembrane region via a hinge, thus anchoring the entire CAR molecule to the cell membrane. Intracellularly, there exists an intracellular domain containing activation signals, which consists of a costimulatory molecular domain and a CD3ζ signaling domain (109, 110).

**Figure 5:**
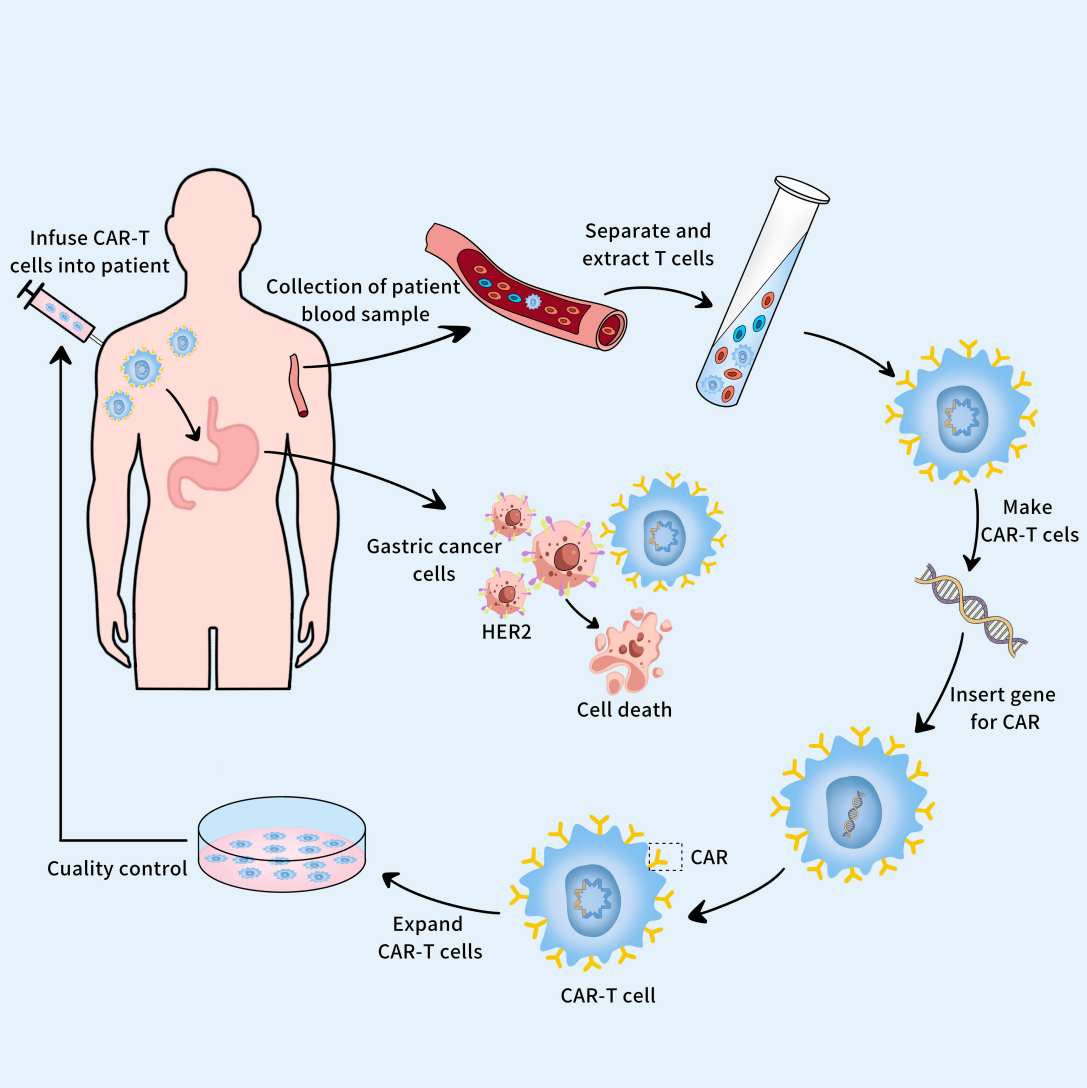
The CAR-T cell therapy and gastric cancer.

Currently, CAR-T cell therapy has achieved remarkable success in treating hematologic malignancies such as B-lymphocytic leukemia and B-cell non-Hodgkin lymphoma (111). In recent years, its potential efficacy in treating solid tumors has also been recognized (112). When applied in the treatment of HER2-positive GC (**Figure 5**), the HER2 antigen-specific activation of expanded CAR-T cells can effectively eradicate HER2-positive GC cells from patients, enhancing treatment efficacy and prognosis (11). The study revealed that CAR-T cell therapy effectively targeted the HER2 antigen on the surface of GC cells in an MHC-independent manner, leading to the apoptosis of tumor cells (113). Compared with controls, CAR-T cell therapy demonstrated potent tumor suppression and cytotoxic abilities in HER2-positive xenograft tumors (113). Additionally, study also demonstrated that CAR-T cells exhibited significant tumor suppression and precise targeting in comparison to untransduced T cells (114). Another study evaluated the safety, feasibility, and clinical efficacy of CAR-T cell therapy in patients with HER2-positive advanced tumors and found that one patient achieved a partial response lasting 4.5 months, and five patients had stable disease with a median progression-free survival of 4.8 months (115). This indicates that CAR-T cell therapy has favorable clinical efficacy. However, during a treatment, a patient with HER2-positive advanced GC developed severe upper gastrointestinal bleeding, suggesting that HER2-targeted CAR-T cell therapy may carry certain risks during the anti-tumor process. Early studies targeting Claudin18.2 and Claudin6 in CAR-T cell therapy for gastrointestinal tumors also show substantial potential (116–118).

CAR-T therapy stands as a revolutionary gene-therapeutic technology, widely employed in cancer treatment, particularly for hematological neoplasms. Although its current application in solid tumors is somewhat restricted, CAR-T cell therapy remains a highly promising immunotherapeutic strategy for solid tumor ablation. CAR-T cells are cells endowed with specific recognition capabilities, generated by modifying autologous T cells. These cells can endure and proliferate within the body over an extended duration, precisely identify, and eradicate cancer cells. By minimizing the risk of immune rejection, they also confer a long-term anti-cancer effect, thereby eliminating the requirement for repeated treatments.Notwithstanding, the clinical utilization of CAR-T cell therapy remains relatively limited. This could be ascribed to its high cost, substantial technical complexity, and the potential onset of severe adverse reactions, such as cytokine release syndrome (CRS) and neurotoxic responses.

As a novel cancer treatment modality, CAR-T cell therapy demonstrates remarkable efficacy and broad application prospects. Nevertheless, issues such as its side-effects and treatment costs still demand further research and resolution. It is expected that in the near future, with the continuous progress of genetic engineering technology, through the optimized design of CAR-T cell therapy, as well as the regulation of tumor burden, antigen expression, and distribution, CAR-T cell therapy will evolve into an economical, safe, and effective treatment option for solid tumors, including gastric cancer.

## 5 HER2 vaccine

A cancer vaccine represents a form of active immunotherapy. It functions by introducing tumor antigens into the patient’s body, thereby activating the body’s immune system. This activation enhances the immune response against the tumor, enabling the body to recognize and eliminate cancer cells (119). Gastric cancer vaccines are a subset of cancer vaccines. Common types of gastric cancer vaccines include cellular vaccines (such as tumor cell vaccines or immune cells), protein/peptide vaccines, and nucleic acid vaccines (DNA vaccines, RNA vaccines, or viral vector vaccines).

HER2 serves as a tumor-associated antigen in gastric cancer and is an excellent target for targeted therapy. The HER2 dendritic cell vaccine can be utilized for the treatment of HER2-positive gastric cancer. One study revealed that 9 patients with unresectable or recurrent HER2-positive gastric cancer achieved partial clinical remission, accompanied by a decrease in tumor markers (CA19-9, CEA) after treatment with the HER2 dendritic cell vaccine. Additionally, 1 patient had a 3-month stable disease period (120). A phase I trial demonstrated that the HER2 dendritic cell vaccine exhibited favorable clinical efficacy in patients with HER2-positive gastric cancer, along with an outstanding safety profile (121). Jung discovered that the BVAC-B vaccine (an autologous B-cell-and monocyte-based immunotherapy vaccine) activated immune cells in patients with HER2-positive gastric cancer, although its clinical efficacy was limited, in the treatment of HER2-positive advanced gastric cancer (122). HER-Vaxx is a B-cell vaccine targeting HER2. In clinical practice, the overall survival (OS) was significantly longer in patients who received HER-Vaxx in combination with chemotherapy compared to those who received chemotherapy alone (13.9 months vs 8.3 months). Moreover, it was found that the levels of HER2-specific antibody IgG induced by HER-Vaxx were significantly correlated with a reduction in tumor volume. HER-Vaxx also inhibited the phosphorylation of the HER2 signaling pathway and promoted ADCC effects (123).

Although there is currently no FDA-approved cancer vaccine for the treatment of gastric cancer available on the market, this field holds substantial potential for development. We eagerly anticipate breakthroughs in combination trials involving multiple vaccines.

## 6 Mechanism of drug resistance to HER2-targeted therapy

Over the past few decades, the landscape of cancer treatment for patients has undergone a remarkable transformation with the emergence of monoclonal antibodies, immune-checkpoint inhibitors, bispecific antibodies, and innovative T-cell therapies. ADC drugs have also brought about a revolutionary change in cancer treatment. Nevertheless, their efficacy remains constrained by drug resistance stemming from diverse mechanisms. These include antigen-related drug resistance, aberrant signaling pathways, incorrect internalization pathways, lysosomal dysfunction, and overexpression of efflux pumps. If these drug-resistance issues can be effectively resolved, it will undoubtedly be a great blessing for cancer patients.

### 6.1 Heterogeneity of HER2 expression

Heterogeneity in HER2 expression implies that, within GC cells, the expression of the HER2 protein varies among different cells or different regions of the same tumor, as well as between primary and metastatic lesions. Heterogeneity of HER2 expression in GC tissues is prevalent, with the incidence of heterogeneity ranging from 30.0% to 75.4% across different studies (124). HER2 expression levels vary significantly across different regions of GC tissues, influenced by microenvironmental factors such as vascular distribution, nutrient supply, and cell-cell interactions. This regional heterogeneity presents challenges in detecting HER2 and other biomarkers for targeted therapy. HER2 heterogeneity is regarded as a poor prognostic indicator in GC, as patients with heterogeneous HER2-positive expression have significantly shorter PFS and OS than those without heterogeneous expression (125). Additionally, it has been demonstrated that HER2 expression heterogeneity is associated with the amplification of other receptor tyrosine kinases (RTKs), such as EGFR, MET, and FGFR2, thereby facilitating resistance to HER2-targeted therapies.

Furthermore, the heterogeneity of HER2 expression between primary and metastatic lesions should not be overlooked. The GASTHER1 study reported that 5.7% of patients with HER2-negative advanced GC had HER2-positive metastases (126). During GC metastasis, GC cells undergo a series of genetic and epigenetic alterations. HER2 expression in metastatic cancer cells can differ from that in primary lesions. In some patients, HER2 expression is low in primary tumors but elevated in metastatic sites. As a result, targeted therapy regimens based solely on HER2 detection in primary lesions may fail to effectively target metastases, potentially compromising treatment efficacy and patient prognosis. Therefore, the European Society of Oncology (ESMO) recommends HER2 testing for metastatic GC, and the American Society of Clinical Oncology (ASCO) recommends HER2 testing for all primary or metastatic GC lesions in patients who can tolerate combination therapy (127).

### 6.2 Loss of HER2 expression

The loss of HER2 expression represents a targeted mechanism of acquired resistance to HER2-targeted therapy. The expression of HER2 on the cell surface of resistant cancer cells is significantly decreased compared to that of cancer cells sensitive to targeted therapy (128). Studies have shown that 69% of GC patients with HER2-positive tumors lose HER2 expression after receiving targeted HER2 therapy, and this secondary loss of HER2 expression is for a major contributor to drug resistance (129). Another study confirmed that29.1% of advanced GC patients treated with HER2-targeted agents experienced a change from HER2-positive to negative status (130). This change diminished the efficacy of targeted HER2 therapy and contributed to drug resistance. It was also found that HER2 heterogeneity increased from 2.9% to 21.9% in metastases, and most patients who became HER2-negative after targeted HER2 therapy also exhibited new heterogeneity. The loss of HER2 expression and changes in heterogeneity are associated with adverse effects of targeted therapy and a shortening of PFS. The factors driving the overall change in HER2 expression remain unclear, and further research on the mechanism is required. It is also recommended that patients who have progressed after first-line targeted HER2 therapy should consider HER2 testing to determine the current HER2 expression status and guide subsequent treatments.

### 6.3 HER2 binding is impaired

The binding of monoclonal antibodies to HER2 is influenced by multiple factors. Among them, deletion of the extracellular segment of the HER2 receptor and molecular masking are potential resistance mechanisms. The p95HER2 receptor, which is a soluble truncated form resulting from the shedding of the full-length p185HER2 receptor, serves as a poor prognostic marker and increases the risk of lymph node metastasis (131). Tumors expressing the full-length receptor have been observed to have a high response to trastuzumab. In contrast, the truncated P95HER2 shows resistance and is also anticipated to lead to T-DM1 resistance because the p95HER2 receptor lacks trastuzumab binding sites (132).Molecular masking may be another contributing factor to resistance. Cells in the tumor or the microenvironment express membrane-associated mucin 4 (MUC4). The interaction between MUC4 and HER2 exerts a steric hindrance effect, inhibiting ADCC. MUC4 masks the binding site on the extracellular segment of HER2, preventing the monoclonal antibody from binding to HER2, which leads to resistance to both trastuzumab and T-DM1. However, silencing MUC4 can restore the sensitivity of tumor cells to T-DM1 (133–135).Moreover, neuromodulins can also impact the sensitivity of ADC drugs. They impair the efficacy of T-DM1 by promoting the heterodimerization of HER2 with HER3 and HER4 (92).

### 6.4 PI3K/AKT signaling pathway

The PI3K/AKT signaling pathway is activated in a variety of tumors and regulates multiple mechanisms, including cell survival, proliferation, growth, metabolism, angiogenesis, and metastasis (136). A The PI3K/AKT pathway, a critical downstream signaling pathway of HER2, plays a pivotal role in the development of drug resistance. This pathway is regulated by multiple factors, such as mesenchymal-epithelial transition factor (MET) amplification, phosphatidylinositol 4,5-diphosphate 3-kinase catalytic subunit alpha (PIK3CA) mutation, and phosphatase and tensin homolog (PTEN) deletions.

#### 6.4.1 MET amplification

Recent studies have shown that MET activation is associated with increased resistance to multiple targeted therapies (137). MET is a tyrosine-kinase receptor present on cell membranes that is often overexpressed or co-amplified in HER2-positive GC and is associated with a lower ORR (138). Previous studies have also revealed that the co-amplification of MET and HER2 results in a significant reduction in OS in GC compared to the overexpression of MET and HER2 alone (139). Further studies have revealed that increased levels of MET and its ligand, hepatocyte growth factor (HGF), contribute to resistance to HER2-targeted therapy by re-stimulating the PI3K/AKT signaling pathway. This resistance can be effectively reversed through the inhibition of MET activity (140). Similarly, the inhibition of MET has been shown to help overcome resistance in afatinib-resistant GC cell lines (141).

#### 6.4.2 PIK3CA mutation

PIK3CA is a proto-oncogene located on human chromosome 3 (3q26.3), and mutations have been identified in a variety of tumors, including breast, endometrial, colorectal, and GC tumors (142–145). PIK3CA mutations can enhance its kinase activity, leading to the sustained activation of the PI3K/AKT signaling pathway, which in turn is associated with the development of resistance to anti-HER2 therapy (142). PIK3CA mutations occur in approximately 4% to 25% of GC patients (146). In addition, there is a significant correlation between PIK3CA mutations and HER2 gene amplification (147), which may provide an explanation for the development of resistance to anti-HER2 therapy.

#### 6.4.3 PTEN loss

PTEN is a tumor-suppressor gene that inhibits tumor invasion and metastasis (148). Genetic mutations are often the main cause of PTEN activity loss, which is closely related to the shortening of PFS and OS and the efficacy of targeted therapy. Studies have shown that PTEN loss occurs in a significant proportion of patients with HER2-positive gastric cancer (GC), with one study reporting a prevalence of 34.5% (149) and another reporting 47.9% (150). At the same time, PTEN is also associated with HER2 overexpression (151), and the deletion of PTEN activity leads to the over-activation of the PI3K/AKT signaling pathway. In PTEN-deficient GC cells, the effect of anti-HER2 therapy on tumor growth inhibition and apoptosis is significantly reduced because PTEN deletion can activate the PI3K/AKT signaling pathway (152), thereby affecting the sensitivity of HER2-targeted therapy. In addition, the down-regulation of PTEN is associated with miR-21, which can negatively regulate PTEN and up-regulate the PI3K/AKT signaling pathway, leading to the emergence of drug resistance (153). This finding is beneficial for addressing the drug-resistance problem of HER2-targeted therapy in some patients and provides a reference for formulating personalized treatment plans.

### 6.5 MEK/ERK signaling pathway

Aberrant activation of the MEK/ERK signaling pathway is closely related to the occurrence and development of various cancers, and this pathway is involved in regulating cell proliferation, differentiation, and apoptosis. Steroid-receptor coactivator (SRC) is a non-receptor tyrosine kinase that can activate multiple downstream signaling pathways, such as MEK/ERK, PI3K/AKT, JAK/STAT, and other signaling pathways (154). SRC expression in HER2-positive tumor cells has been found to be consistent with HER2 expression (155), and evidence suggests that SRC may act as a link between EGFR and MET (156). These findings imply that SRC-mediated activation of downstream signaling pathways could play a role in the development of resistance to HER2 therapy. One study demonstrated that in HER2-positive breast cancer, SRC activation drives trastuzumab resistance, and treatment with SRC inhibitors can effectively reverse this resistance (157). Additionally, the use of Bosulif, an SRC tyrosine-kinase inhibitor, was able to block downstream signaling, thereby restoring sensitivity in gastric and biliary cancer cell lines that were previously resistant to Trastuzumab (158).

### 6.6 Wnt/β-catenin signaling pathway

The Wnt/β-catenin signaling pathway is essential for regulating cell proliferation, differentiation, and survival. It is influenced by a variety of factors, such as Wnt protein family members, precursor cytokines, genetic variations, microRNAs and other regulatory elements (159). Dysregulation of this pathway is associated with numerous diseases, such as cardiovascular and lung diseases, and its abnormal activation is also closely related to tumorigenesis, progression, and poor prognosis (160). β-catenin can directly bind to HER2 and promote the phosphorylation of HER2 at Y877 and Y1248 and is also capable of inducing resistance to Trastuzumab treatment by promoting the HER2-SRC interaction (161).The study found that Wnt3A, FZD6, and CTNNB1 were up-regulated in trastuzumab-resistant cells, while GSK-3β was down-regulated (162). By modulating CTNNB1, this signaling pathway can achieve a reversal of the resistance phenotype. Another study also demonstrated that the Wnt/β-catenin signaling pathway promotes the development of drug resistance during targeted therapy by regulating the stemness and EMT phenotype of GC cells (163). These findings suggest that targeting the Wnt/β-catenin signaling pathway may overcome drug resistance in HER2-positive GC-targeted therapy.

### 6.7 HER2-SHCBP1-PLK1-MISP signaling pathway

SHCBP1 is a SHC1-binding protein that is a downstream effector protein of HER2 and plays an important role in cell signaling mechanisms. HER2 interacts with SHCBP1 to activate PLK1 and regulate the cell cycle and mitotic progression. Numerous studies have shown that SHCBP1 is up-regulated in various types of tumor tissues (164), and the expression level of SHCBP1 in breast cancer is closely related to the expression levels of GC and HER2 (165, 166). In addition, resistance to anti-HER2 therapy has been linked to the downstream signaling pathway of PLK1, and targeting PLK1 can enhance drug sensitivity (167). However, research on the role of the PLK1 signaling pathway in GC treatment remains limited. HER2 can activate the SHCBP1-PLK1-MISP signaling pathway, promote cell mitosis, reduce the sensitivity of HER2-positive GC cells to Trastuzumab, and TFBG sensitizes GC to Trastuzumab treatment by blocking the SHCBP1-PLK1-MISP axis (168).

### 6.8 Wrong way of internalization

When an ADC drug binds to the target antigen, it enters cancer cells via endocytosis. Endocytosis mechanisms include clathrin-mediated endocytosis, caveolin-mediated endocytosis, and clathrin-caveolin-independent endocytosis. Among them, clathrin-mediated endocytosis is the most frequently adopted pathway by ADCs (169). Once endocytosis is impaired, ADC drugs cannot function properly. Thus, impaired endocytosis is regarded as a mechanism of ADC resistance. EndophilinA2 (encoded by SH3GL1) is a scaffold protein involved in endocytosis. In HER2-positive breast cancer models, EndophilinA2 promotes HER2 internalization and enhances the sensitivity of breast cancer cells to trastuzumab treatment. Targeting the SH3GL1 gene to inhibit EndophilinA2 expression results in reduced internalization and significantly inhibits T-DM1-mediated cytotoxicity (170). In T-DM1-resistant gastric cancer N87 cell lines, caveolin-mediated endocytosis was found to be significantly enhanced. This might be because caveolin is not conducive to drug transport to lysosomes. However, knocking down caveolin did not restore T-DM1 sensitivity (171). Another study indicated that upregulating the expression of caveolin improved the activity of T-DM1 in different cell lines (172). Additionally, hypoxia-induced translocation of caveolin from vesicles to plasma membranes has been proposed as a possible mechanism for the reduced internalization of trastuzumab in the hypoxic microenvironment of HER2-positive breast cancer (173). In summary, the role of caveolae-mediated endocytosis in T-DM1 resistance remains controversial and may be related to the dominant endocytic pathway of the target antigen in certain cell lines.

### 6.9 Lysosomal dysfunction

The ADC drug target molecule binds and enters the cell through endocytosis. Once it reaches the lysosome, the cytotoxic agent in the ADC drug is released by chemical or enzymatic cleavage. The degradation of ADCs depends on the acidic lysosomal environment and active lysosomal enzymes. It has been discovered that T-DM1 accumulates in lysosomes in cells that develop resistance to T-DM1. The proteolytic activity in these drug-resistant cells is lower than that in sensitive cells. This is due to an increase in lysosomal pH, which inhibits proteolytic enzyme activity, resulting in T-DM1 not being adequately cleaved to release cytotoxic agents (174). Moreover, the acidic environment required for ADC degradation in lysosomes is maintained by V-ATPase, a proton pump that regulates lysosomal acidity (175). Low V-ATPase activity was detected in T-DM1-resistant N87 cell lines. Inhibition of V-ATPase activity in T-DM1-sensitive N87 cells with bafilomycin A1 induced resistance to T-DM1, suggesting that abnormal V-ATPase activity reduces T-DM1 metabolism, leading to T-DM1 resistance (176). Therefore, V-ATPase activity in lysosomes can be used as a novel biomarker to predict T-DM1 resistance. The cytotoxic agents in ADC drugs exert cytotoxic effects during their transport from lysosomes to the cytoplasm through lysosomal transporters. SLC46A3 belongs to the solute carrier (SLC) transporter family, which is a transporter on the lysosomal membrane. Silencing of this protein leads to the accumulation of catabolites in lysosomes, ultimately resulting in drug failure (177).

### 6.10 Overexpression of drug efflux pump

ATP-binding cassette transporters (ABCs) are transporters located on cell membranes and play a crucial role in drug absorption, distribution, metabolism, and excretion (178). Their overexpression causes the extracellular pumping of cytotoxic drugs from the cell, inhibiting the cytotoxic effects of anticancer drugs. This is one of the main causes of multidrug resistance in human malignancies (179). Since many payloads are substrates for ABC, upregulation of ABC can lead to an increase in the active efflux of payloads (180), thereby protecting tumor cells from cytotoxic damage and causing ADC resistance. The ABC transporter family consists of 48 proteins, including ABCB1 (also known as MDR1/P-glycoprotein), ABCG2, ABCC1, ABCC2, and others (181). ABCB1-mediated efflux is the most common in clinical resistance, and ABCB1 has been shown to be a major driver of resistance to vc-MMAE-based conjugates (vc: valine-citrulline, MMAE: tubulin inhibitors) (182). Another study demonstrated that the expression of the ABC transporter ABCG2 or ABCB1 leads to DXd/SN-38 efflux, causing the drug-resistance problem in DXd/SN-38 ADCs (183). The use of ABCG2 inhibitors and ABCB1 inhibitors, respectively, on ASPC-1 and H2170 cells with high ABCG2 expression and HCT15 cells with high ABCB1 expression can enhance the sensitivity of cancer cells to DXd/SN-38.

### 6.11 Tumor metabolic reprogramming and tumor microenvironment

Metabolic reprogramming of tumor cells is an important manifestation of their adaptation to the external environment and plays a crucial role in tumor growth, proliferation, metastasis, and drug resistance. Numerous studies have shown that metabolic reprogramming has been observed in trastuzumab-resistant HER2-positive GC, including glycolysis, amino acid metabolism, and lipid metabolism (184).

Glycolysis plays a critical role in tumor metabolism, with tumor cells exhibit higher glycolytic activity compared to normal cells. In HER2-positive GC, fructose-6-phospho-kinase 2 activates the glycolytic pathway, contributing to the development of trastuzumab resistance Targeting PFKFB3 (a key enzyme for glycolysis) can increase sensitivity to anti-HER2 therapy (185). It was found that the related transporters and metabolic enzymes involved in glutamine catabolism were significantly increased in drug-resistant cells, and glutaminase 1 (GLS1) was the most elevated (186). Lipid metabolism is also involved in the anti-HER2 therapy process, and fatty acid synthase is significantly expressed in drug-resistant cells. The combined treatment of HER2 and fatty acid synthase has achieved a favorable anti-tumor effect (187).

The tumor microenvironment (TME) is composed of a variety of immune cells and proteins, and its main function is to promote tumor progression (188). The immune cells within the TME have a dual role, both inhibiting tumors and participating in tumor angiogenesis, immunosuppression, and cell proliferation through cytokine secretion (189). These complex and multiple roles of immune cells in the TME tend to promote tumor progression and enhance resistance to drug therapy. Studies suggest that HER2 signaling may be involved in the regulation of immune cell activation in the TME, which inhibits immune cell activation in the TME by inhibiting interferon-gene-stimulating factor signaling in GC cells, resulting in drug resistance (190). Macrophages in the TME are closely related to tumor progression. Studies have shown that GC cells promote GLS1 expression and the release of GLS1-containing microvesicles into the TME through the CDC42/NF-κB signaling pathway (186). These microvesicles promote macrophage polarization to the M2 phenotype and pro-angiogenic functions, leading to acquired trastuzumab resistance in HER2-positive GC cells.

The mechanism of drug resistance in HER2-targeted therapy has been relatively well-elucidated. However, in clinical practice, it remains infeasible to prevent the emergence of drug resistance through technical detection methods. This could be attributed to several reasons. First, the development of drug resistance may be driven by multiple mechanisms, such as the activation of drug-efflux pumps, target mutations, and bypass activation. As a result, it is arduous for a single biomarker or mechanism to comprehensively account for the overall drug-resistance phenotype. Second, it may be associated with treatment-related stress. The mechanism of drug resistance can change dynamically in response to treatment-induced stress, such as tumor evolution or adaptive mutations. Moreover, clinical samples are typically collected at a single time-point, which makes it challenging to capture this dynamic process. Third, it may also be related to factors such as the patient’s immune status, gut microbiota, or metabolites. Regrettably, these factors are often neglected in current clinical practice.

## 7 Conclusion and prospects

GC ranks among the most prevalent malignancies worldwide, being characterized by high morbidity and mortality rates. A substantial number of GC patients are often diagnosed at the middle-and advanced-stages. This makes treatment far more challenging, leads to a poor prognosis, and results in a low survival rate. The role of human epidermal growth factor receptor 2 (HER2) in GC has been well-established. Targeting HER2 has significantly improved the outcomes of both early-and late-stage HER2-positive GC patients. Nevertheless, drug resistance is a common issue in current GC treatments, whether involving traditional chemotherapy drugs or some of the targeted-therapy drugs already in use.

ADCs are characterized by more precise targeting and stronger cytotoxicity, enabling them to more effectively recognize and eliminate GC cells. Combination-therapy regimens can exert drug synergy through different mechanisms of action, interfering with the growth, proliferation, and metastasis of tumor cells at multiple levels, thereby enhancing the prognosis of patients. However, problems such as adverse reactions and drug resistance still persist. Thus, it is crucial to re-optimize the design strategy, explore the development of bispecific-antibody ADCs to enhance efficacy and safety, identify new predictive biomarkers, and achieve precise and individualized treatment. For first-line ADC combination-therapy regimens, if the tumor can be effectively controlled, it can reduce the occurrence of tumor-related symptoms, thereby improving the patient’s quality of life. Additionally, patients are in relatively better physical condition and more tolerant of new treatment options during first-line treatment compared to second-line or subsequent treatments. The research and development of new ADCs and combination regimens as first-line therapies can drive innovation and development in the field of GC therapeutics. Through continuous exploration of new drug combinations and treatment models, more treatment options can be provided for GC patients, and references and ideas can also be offered for the treatment of other malignant tumors.

As an innovative and promising ablative immunotherapy for solid tumors, CAR-T therapy is gradually emerging, attracting the attention of numerous researchers and clinicians. CAR-T cell therapy has achieved remarkable results in the treatment of hematologic tumors, significantly improving the survival of some patients. Many experts and scholars predict that in the near future, CAR-T cell therapy is expected to overcome many difficulties and become a safe and effective treatment modality for various solid tumors, including GC. However, the emergence of drug resistance has gradually become a key factor impeding its further development and affecting patient prognosis. Tumor cells are highly heterogeneous and can evade CAR-T cell attack through a variety of complex mechanisms. For example, tumor cells may downregulate or alter the antigen expression on their surface, making it difficult for CAR-T cells to recognize them; they may also interfere with the activity and function of CAR-T cells by secreting some inhibitory cytokines. The emergence of these drug-resistance phenomena greatly reduces the long-term effectiveness of CAR-T cell therapy, leaving some patients still at risk of tumor recurrence and disease progression after treatment.

Looking ahead, the treatment of HER2-positive GC patients holds great potential. In terms of exploring new targets, researchers will focus on the molecules on the surface of tumor cells that have not been fully studied and the key regulators in the tumor microenvironment, aiming to identify new and more specific targets to lay the foundation for the development of more efficient therapeutic drugs. Besides traditional small-molecule targeted drugs and antibody drugs, drugs based on new mechanisms of action, such as bispecific antibodies, improved ADCs and antibody neodegrader conjugate (AnDC), are expected to exhibit unique advantages in the treatment of HER2-positive GC. In the exploration of new models, in addition to the combined application of various treatment methods carried out so far, personalized treatment models will also be considered based on the individual characteristics of patients, such as genetic background, lifestyle habits, and underlying diseases. This can realize the transition from “one-size-fits-all” treatment to precise and individualized treatment, bringing unprecedented breakthroughs to the treatment of HER2-positive GC patients.
